# Pediatric Obesity: Looking into Treatment

**DOI:** 10.3390/nu1020197

**Published:** 2009-11-30

**Authors:** Angelo Pietrobelli, Simone Rugolotto, Paolo De Cristofaro, Marcella Malavolti

**Affiliations:** 1 Pediatric Unit, Verona University Medical School, Verona, 37134 Italy; Email: rugolotto@hotmail.com; 2 Applied Dietetic Technical Sciences Chair, Modena and Reggio Emilia University, Modena, 40100 Italy; Email: marcella.malavolti@unimore.it; 3 Centro Regionale di Fisiopatologia della Nutrizione ASL Teramo, Giulianova 64022 Italy; Email: pdecristofaro@gmail.com

**Keywords:** children, adolescents, nutritional assessment, cardio-vascular risk factors, adiposity, physical activity, lifestyle changes

## Abstract

Prevalence of pediatric obesity continues to rise worldwide. Increasing the number of health care practitioners as well as pediatricians with expertise in obesity treatment is necessary. Because many obese patients suffer obesity-associated cardiovascular, metabolic and other health complications that could increase the severity of obesity, it is fundamental not only to identify the child prone to obesity as early as possible, but to recognize, treat and monitor obesity-related diseases during adolescence. This short review outlines the treatment of pediatric obesity that may have applications in the primary care setting. It examines current information on eating behavior, sedentary behavior, and details studies of multidisciplinary, behavior-based, obesity treatment programs. We also report the less common and more aggressive forms of treatment, such as medication and bariatric surgery. We emphasize that health care providers have the potential to improve outcomes by performing early identification, helping families create the best possible home environment, and by providing structured guidance to obese children and their families.

## 1. Introduction

The causes of pediatric obesity are both genetic and environmental [1]. Twin studies have clearly demonstrated the genetic link, and the discovery of hormones that influence appetite, satiety and fat deposition provide the underlying metabolic mechanisms giving rise to different physiologic risks of obesity [1,2]. To allow the development of obesity, both genetic and environmental factors must influence one or more components of energy balance. At the population level, the increase in prevalence is too rapid to be explained by a genetic shift, but it is more related to changes in eating and physical activity, behaviors that alter the balance of energy intake and energy expenditure [2,3,4]. 

Epidemiologic evidence supports the theory that the relation between obesity and disease risk begins early in life, and those risk factors for disease track, or remain at a similar level, with advancing age, growth, and development [1,2,3,4]. 

The classification of obesity and overweight are age- and gender-specific and are defined on the basis of Body Mass Index (BMI, calculated as weight in kilograms divided by the square of height in meters, kg/m^2^) normative values that were established when the distribution of BMI values was constant [5]. The definition of overweight among children is a statistical definition based on the 2,000 Centers for Disease Control and Prevention growth charts for the United States. Overweight is defined as at or above the 95^th^ percentile of BMI for age. At risk for overweight is defined as at or above the 85th percentile, but less than the 95th percentile of BMI for age [5]. The increase in obesity prevalence is therefore measured against a stable cutoff point, the 95^th^ percentile BMI for gender and age.

On the basis of measured height and weight from nationally representative samples of US children assessed approximately every five years, obesity prevalence has increased from 5% in 1963 to 1970 to 17% in 2003 to 2004 [6]. Recently using a nationally representative sample, it was reported in USA that the prevalence of overweight has increased, among children 6 to 24 months of age, from 7.2% in 1976–1980 to 11.6% in 1999–2000 [2]. 

Overweight and obese subjects have a greater risk of developing cardiovascular risk factors, such as hypercholesterolemia, hypertension, and hyperlipidemia. Overweight subjects also have a higher risk of developing type 2 diabetes, obstructive sleep apnea, orthopedic complications, renal diseases, and metabolic syndrome [2,3,6]. Fat tissue, once considered as an inert depot for energy storage, is now seen as metabolically active. Fat cells secrete agents that regulate a host of physiologic processes directly related to carbohydrate and fat metabolism and the development of cardiovascular disease and type 2 diabetes [3,4]. Being overweight also has a negative impact on subjects’ psychological state that could be a reflection of body dissatisfaction and binge-eating behaviors [2]. 

Although pediatricians are concerned with the problem of pediatric obesity, most feel unprepared and ill equipped to address it [7]. A survey of pediatricians, dieticians, and pediatric nurse practitioners, confirm that pediatricians do indeed face many challenges in treating childhood obesity [8]. Most pediatric primary care providers are not trained to offer the extensive counseling required to treat obesity. Most have insufficient time to dedicate to the obese child. Pediatricians may also be frustrated by what they see as insufficient patient motivation and lack of parental concern [7]. 

Because the high and rising prevalence of obesity among children is a key concern a strong stance needs to be taken. In order to improve the quality of health care and to minimize cost it is important to investigate and standardize pediatric obesity prevention and treatment and to adapt to it to both social and cultural aspects. This approach will make it easier for health care personnel to help patients and families. 

The enormous cost of obesity and its complications, particularly when initiated in childhood, emphasizes the importance of treating childhood obesity at its inception [9,10]. Surprisingly, little evidence-based, high quality research has been carried out on identifying interventions to treat childhood obesity. This review will provide a synopsis of treatments that have been evaluated in appropriate studies. It will put particular emphasis on novel approaches and as such will consider papers mainly published in the last five years. The worsening obesity epidemic makes it critical to continue examining and developing new and more effective treatment strategies with the goal of a coordinated approach involving government, communities, and health-care providers to offer a continuum of population-based interventions, focused screening, and personalized multidisciplinary interventions for the obese child and their family [10,11,12]. 

### Treatment choice

Many different treatments have been investigated, including diet, exercise, surgery and medication. None have been found to be effective as a sole treatment. Several studies have been performed in more lab-oriented setting, while others have focused on free living conditions [8,9,11,12,13]. It is clear that treatments need to be both affirmative and long lasting. Single physical treatments are ineffective as they fail to take into account related psychological factors and the lifelong influence of genetics [9,10,13]. The necessity of implementing “chronic” treatment results from an increasing knowledge of genetics, where an inherited susceptibility to develop obesity has been identified. Thus, a lifelong treatment program of exercise and diet is required. 

Ultimately, the goal of obesity treatment is the improvement of long-term physical health through permanent changes towards a healthy lifestyle. Implicit in this approach is that clinicians, patients and parents work together. In fact, the commitment of parents and other caregivers in helping a child to develop healthy habits to treat obesity is fundamental [8,13]. 

### Proposition for a treatment program

Based on recent reports [8,11,12,13] on prevention, assessment and treatment of obesity a step by step or staged approach for weight management in the pediatric population is highly recommended. The recommended care process according to the Expert Committee of American Academy of Pediatric is divided into four stages, (1) prevention with the goal of healthy lifestyle changes, (2) structured weight management, (3) comprehensive multidisciplinary intervention, and (4) tertiary care intervention/ treatment [13]. 

Stage 1, the prevention stage, provides an initial treatment intervention useful for patients 2 to 18 years of age that are overweight or obese. The interventions should be based on the family’s readiness to change and include the following aims: (i) consumption of ≥5 servings of fruits and vegetables per day, (ii) reduction or elimination of sweetened beverages, (iii) reduction of TV or video games (“screen time”) to 2 hours per day with no television in the child’s bedroom, and (iv) at least one hour of physical activity per day. Patients and family members also have to follow set eating patterns. Specifically: (i) eating breakfast daily, (ii) limiting meals outside home, including fast food venues, (iii) eating family meals once a day. Cultural difference related to eating behaviors must be taking into consideration [9]. The goal should be weight maintenance with growth that results in a BMI reduction as age and height increase. If no improvement of BMI reduction occurs after approximately 6 months, advancement to Stage 2 is highly recommended, keeping in mind patient/family readiness to change [9,13]. 

Stage 2, structured weight management, recommends the following: (i) development of a plan with a balanced diet and small amounts of energy-dense foods, (ii) provision of structured daily meals and appropriate snacks (*i.e.*, breakfast, mid-morning snack, lunch, mid-afternoon snack and dinner), (iii) ≥1 hour of structured active play per day, (iv) ≤1 hour of daily “screen” use, (v) increased behavior monitoring of all the structured activities (*i.e.*, diet, activity, screen time use), (vi) reinforcement for achieving targeted behavior goals, but no mention of weight goals to the patient, particularly adolescents, should be made . In Stage 2, for subjects in the 85^th^–94^th^ percentile with health risks, the goal should be weight maintenance resulting in a BMI decrease as age and height increase. In this stage, in subjects in the 95^th^–99^th^ percentile it is suggested weight loss should be gradual (0.5 kg/month), while in subjects >99^th^ percentile, weight loss should not exceed approximately 1 kilogram per week. If no improvement in BMI status occurs in 3 to 6 months, the patient needs to be advanced to Stage 3. Ideally in Stage 2, a dietician with expertise in childhood obesity should provide the nutrition counseling as well as an exercise physiologist to provide physical activity counseling both in conjunction with the primary care provider. Additional training in motivational interviewing/behavioral counseling, monitoring and reinforcement, family conflict resolution could help primary care providers to implement treatment [9,13,14]. Parents should be involved in behavioral modification and sometimes a referral to a physical activity program for the entire family may be necessary to help some families to develop an active lifestyle [9,13]. 

Stage 3, provides a comprehensive multidisciplinary intervention, eating and activity goals remain the same as in Stage 2. Activities within this category should also include the following: (i) planned negative energy balance achieved through structured diet and physical activity, (ii) structured behavior modification program including the development of short-term goals, (iii) provision of training for the whole family to improve the home environment, (iv) frequent clinic visits, with weekly visits for the first 8 to 12 weeks [13]. Systematic, specific and rigorous body composition assessment should be conducted at baseline and at specific intervals in order to control changes in fat and fat free mass development [15]. Because BMI is a surrogate measurement of adiposity, waist circumference should be used to detect visceral adiposity and subsequent cardio vascular risk factors. [4,8,13,15]. Bioimpedance analysis (BIA) should also be used to estimate lean and fat mass [15]. For Stage 3, the goal should be weight maintenance or gradual weight loss until BMI is <85^th^ percentile [13]. For implementation of the intervention, comprehensive treatment should be provided by a multidisciplinary obesity care team, including psychologist, registered dietician/nutritionist, exercise physiologist [9,13,15]. The primary care provider should continue to monitor medical issue and maintain a strong supportive alliance with the family [13].

Stage 4, tertiary care intervention/treatment should be used to only a limited extent in the pediatric population. It is appropriate for severely obese adolescents who have been unable to decrease their adiposity and reduce their morbidity risks [13]. Before entering the Stage 4 intervention patients should have the maturity to understand the possible associated risks. Patients should be willing to maintain physical activity, to follow a diet and to participate in behavior monitoring. Stage 4 should include referral to a pediatric tertiary management center that operates with a designed protocol that considers meal replacement, very-low-energy diet, medication and ultimately bariatric surgery [13]. A restrictive diet should be used as a first step in a childhood weight management program, followed by a mildly restrictive diet. Stage 4 considers the use of drug treatment as well as surgery [2,8,13]. For implementation of Stage 4, the multidisciplinary team should have expertise in childhood obesity and its co-morbidities, with patient care being provided by a physician, nurse practitioner, a registered dietitian, a behavior counselor, and an exercise specialist. 

In conclusion, the role of family in weight control programs cannot be emphasized strongly enough. Parents can serve as role models, authority figures, and “behavioralists” to mold their children’s eating and activity habits [8,10,13]. Clinicians can influence children’s habits indirectly by teaching and motivating parents to use their authority effectively. For young children the clinicians should focus their attention on parenting behavior. With adolescents, due to increasing independence, clinicians should discuss healthy behaviors directly with them, but encourage parents to make the home environment as healthy as possible [8,16].

### Why do we need new treatments?

Many different treatments for obesity have been investigated, including diet, exercise, surgery and medication. None have been found to be effective as stand alone treatments. It is clear that treatment needs to be affirmative and long-term. The necessity for chronic treatment is more widely recognized due to increased knowledge of genetics, where many obese patients have an inherited susceptibility to develop obesity. Thus, obese patients need lifelong treatment and not merely a short period of exercise or a diet program [8,9,11,12]. When a patient is identified as having an inherited predisposition to obesity, an extremely healthy lifestyle is necessary to maintain normal body weight [1,9,11]. Below a number of strategies in the treatment of childhood obesity are outlined. 

### Basic approach – school-based treatments

At school behavioral therapy can be used including behavior modification, nutrition modification and physical activity. Parents and school personnel should all be involved. Implementing the treatment at school promotes a healthy lifestyle to all children and it can be a fruitful approach. However, long term follow-up is not easy in such studies because so many variables have to be controlled for. This approach has been reviewed but with no single “specific” program being identified as better than others and for this reason, no recommendation could be made [8,9]. Interventions must consider the powerful influences of the social context on treatment approaches and the theoretical basis for their use. Healthy lifestyle education should be delivered to students in structured, educational settings with peer support and teacher guidance. It is within the school setting that children are socialized to accept the standards and values of their society and is an ideal setting for promotion of new health behaviors [17].

### Early treatment

Public health researchers have traditionally described three types of prevention for overweight and obesity: primary, secondary and tertiary prevention [8]. Primary prevention is where the medical condition is prevented through early and aggressive intervention. Ideally, intervention completely preempts or interrupts the emergence of the condition even before its early stages. Thus, primary prevention of pediatric obesity requires early-life detection of excessive adiposity or the identification of genetic markers to target individuals who will probably become obese if left untreated [1,8]. 

Treatment that starts before the major peak incidence of pediatric obesity should show positive results. It is similar to school based treatment, but differs because it is directed at obese children [8,9,10,13]. Such early treatment could prevent severe cases of obesity in adulthood. The primary goal of obesity treatment is improvement of long-term physical health through permanent healthy lifestyle habits [8,13]. The establishment of permanent healthy lifestyle habits is a good outcome, regardless of weight change, because of the long-term health benefits of these behaviors.

### Behavior therapy

The program is based on the concept that obesity is a “learned disease” that can be treated by “re-learning”. The therapy is based on the concept of bad eating habits in which an insufficient control of stimulus or rewarding behavior results in increased food intake. These habits can be broken down into small sequences. Parents are regarded as a reinforcement of the subject’s eating habits. The purpose of the behavior assessment is twofold. The first goal is to identify the child’s dietary and physical activity behaviors that promote positive energy balance and that are modifiable [8,13,18]. The second goal of the patients together with their family is to change some and/or all these behaviors [8,13,18]. The clinicians should work with the family to target behavior changes that are possible. Because comprehensive dietary and physical activity assessment are impractical expert committees recommend an assessment of behaviors that have evidence for association with energy balance and that are modifiable [8,13]. 

For eating behavior assessment it is fundamental to address frequency of eating outside the home, the number of sugar-sweetened beverages consumed per day, portion sizes, frequency of breakfast, the number of fruit and vegetables and the number of meals and snacks consumed each day as well as consumption of foods that are high in energy density. For physical activity assessment it is important to know the time spent in moderate physical activity, routine daily activities and sedentary behavior. When inappropriate dietary and physical activity behaviors are detected, the primary care provider should provide support and guidance for both healthy eating and activity habits according to recommendations [2,7,8,11,13]. It is important to achieve the targeted behaviors in steps. This is particularly true for physical activity, starting with 15 minutes physical activity per day and so on up to 60 minutes. 

### Cognitive behavior therapy

This therapy has been used recently in the treatment of obesity usually combined with behavioral therapy. The combination is based on the assumption that, through practice and reward, changes in key areas of children’s cognitive processing will result in behavior changes [9]. 

The family is regarded as basic to the child’s psychological development and a major factor influencing the child’s quality of life. Family therapy in treating obese subjects in conjunction with healthy diet and physical activity has shown obesity reduction in older teenagers if treatment is started as early as possible [9]. In practice, family therapy could be defined as follows: using the encounter with a family to improve the obese member’s health by observing and analyzing interaction between family members, and also with therapists, and improving their ability to use their own resources [18].

### More aggressive interventions

#### Weight loss medications in the treatment of pediatric obesity

The use of weight loss medications in adult obesity treatment should be considered for intensive regimens. Pharmacotherapy alone has not proved to be an effective obesity treatment. Medication used in conjunction with lifestyle modification produce an average weight loss of 5% to 10%, which typically plateau at 4 to 6 months of therapy, after which weight regain may occur [8,11,13]. Few guidelines are available regarding the use of weight loss medication in the pediatric population. Recently Freedman and colleagues [19] using cross-sectional and longitudinal analyses to assess risk factors, concluded that 99^th^ percentile of BMI for age may be an appropriate threshold for identifying children and adolescents who are at very high risk for biochemical abnormalities and are ultimately candidates for pharmacotherapy [19]. Presently among the approved drugs for adults only 2, orlistat and sibutramine, have been approved for limited use in pediatric patients. The Food and Drug Administration has approved sibutramine for patients’ ≥16 years of age and orlistat for patients’ ≥12 years of age.

*Sibutramine*, an appetite suppressant, is a non-selective reuptake inhibitor, mainly of serotonin and norepinephrine but also blocks dopamine re-uptake [8,11,13]. A 1-year, multi-center study on 498 adolescents 12 to 16 years of age found that those who received sibutramine plus behavior therapy lost significantly more weight than did those who received a placebo and behavior therapy [20]. Subjects on the sibutramine group lost an average of 6.35 kg during the study, whereas those in the placebo group gained 1.8 kg. The subjects in the drug group also showed significant decreases in insulin as well as triglyceride levels. Serious side effects, such as hypertension and tachycardia were reported in 19 out of 43 adolescents. Godoy-Matos and colleagues studied the safety and efficacy of sibutramine in a double-blind control study in 60 obese adolescents and found an average weight loss of 8.1 kg at 6 months, but did not find any changes in blood pressure [21]. Although, all investigators involved in these trials, concluded that more research is required to determine long term efficacy and safety of this drug in adolescents [20,21]. In future tests using sibutramine in obese adolescents different strategies should be tested such as the introduction of sibutramine after a period of weight control with lifestyle changes in order to reduce the potential for side effects such as hypertension. 

*Orlistat* is a reversible lipase inhibitor. It binds lipase in the lumen of the stomach and cuts intestinal fat absorption by up to 30%. The side effects of orlistat are abdominal cramp and flatus [22]. The most troubling side effects are oily bowel movements, flatus with discharge, and oily spotting on underwear caused by unabsorbed fat in the feces [21]. Orlistat does not inhibit other intestinal enzymes and it is minimally absorbed and exerts no effect on systemic lipases [22]. Because it can interfere with the absorption of fat-soluble vitamins, patients taking the drug must also take a daily supplement [8,13,22]. A multi-center, one-year randomized, placebo-controlled trial in 539 obese adolescents 12 to 16 years of age found that those who used orlistat lost weight and had significantly greater reductions in BMI (−0.55 kg/m^2^), whereas those taking a placebo showed a slight increase in BMI (+0.31 kg/m^2^). Changes in waist circumference was found in the treatment group (−1.33 cm) while the placebo group showed an increase (+0.12 cm, p < 0.05). Although, no significant differences in blood glucose or lipid levels was seen in the two groups [22]. The explanation for the failure of lipid and glucose levels to improve could be that the body weight loss was too small to change metabolic risk factors [22]. This study was not able to identify how much BMI must be reduced to provide health benefits and cardio-vascular risks reduction in children and adolescents. 

More than 120 potential drugs for treatment of obesity are currently in various stages of research, but presently no agent that treats obesity as a single therapy is available. A lot is known regarding the molecular mechanisms regulating body fat and the central regulation of energy balance. In this regard it is possible to develop more effective medication. Although medication should be used only as part of a multimodal weight management approach that includes diet, physical activity and behavior modification. 

#### Bariatric Surgery

Severe obesity has proved difficult to treat through diet and lifestyle changes even with the addition of pharmacotherapy. The increased use of bariatric surgery to treat adult morbid obesity and associated co-morbidities has led to interest in using this therapy in adolescents. Usually, in adults surgery is performed only in patients that are severely obese (BMI > 40) or when they have a BMI > 35 together with severe obesity-related health complications [11]. Bariatric weight loss procedures can be divided into three categories, that is, malabsorptive, restrictive and a combination of the two. Combination procedure such as Roux-en-Y gastric bypass, restrict food intake and reduce/limit the amount of nutrients the body absorbs. Gastric bypass procedures are the only form of bariatric surgery currently approved for use in adolescents. Sugerman and colleagues reported retrospective data regarding surgery procedures in adolescents and found significant weight loss with subsequent resolution of most co-morbid conditions [23]. Apovian and colleagues reviewed data of weight loss surgery in adolescents between 1980 and 2004. They found a durable weight loss for the majority of the patients. Although, they found important post surgery complications and mortality rates [24]. Nadler and colleagues showed in 53 obese adolescents that adjustable gastric banding represents an effective treatment strategy for excess weight at 18 months [25] and at 2 years of follow-up [26]. 

Bariatric surgery generally leads to substantial weight loss and improvement in medical conditions. However, peri-operative risks, post-procedure nutritional risks, and the need for lifelong commitment to altered eating make this approach unattractive or inappropriate for adolescents [8,13]. After surgery it is fundamental to maintain lifelong medical supervision to ensure optimal post-operative weight loss, and ultimately overall health. 

#### Proposition for a practical treatment program

The recommendations below are not yet “approved”, but have been extrapolated from guidelines and previously published papers [8,9,11,12,13]. Children should not have any signs of other diseases; usually overweight and obese subjects are tall for their age and gender. Basic pediatric evaluation should include blood pressure measurement, lipid profile control and thyroid function needs to be checked. 

##### Age 0–3 years

No action in particular related to weight loss is recommended.

##### Age 4–10 years

In children with a BMI > 30 and both parents with a BMI < 30. Firstly, it is fundamental to exclude syndromic obesity. When this is excluded, it is important to ascertain the dietary intake of the child, and provide guidance to the parents with regard to fat intake, reducing the fat content of the diet to 30% energy around 1500 kcal a day. In children with a BMI > 30 with one or both parents with a BMI > 30. The child must be carefully evaluated by the pediatrician with regard to the child’s growth chart and encouraged to participate in sport and exercise. A healthy lifestyle approach involving the entire family is needed with clear goals provided. 

##### Age 10–16 years

At this age puberty occurs together with changes in hormonal profiles. In a patient with a BMI between 25 and 30, parents should be advised to reduce the fat content of the diet to 30% energy and the child encouraged to participate in sport and exercise. In patients with BMI > 30 they must be referred to a pediatrician with strong recommendations to modify the lifestyle of the family as a whole including participation in sport and exercise.

## 2. Discussion

The key to successfully treating childhood overweight and obesity ultimately lies in developing and funding a targeted research agenda. At the top of that agenda should be basic research into the biology and physiology of appetite regulation during each developmental stage of childhood. Research should also focus on the mechanisms that regulate body fat distribution during adolescence as well as the influence of gender and ethnicity on body composition and fat differentiation [1,3].

The lack of a clear program to address the precise needs of overweight and obese children has emerged in this review. Several gaps in our knowledge are of concern because (a) the pre-school years may be a critical period for the prevention of obesity as indicated by the association of adiposity rebound and obesity in later years; and (b) although the growing prevalence of obesity affects males and females equally, males may be more vulnerable to associated health risks such as cardiovascular disease [27]. Other issues include a limited number of interventions in home and community settings and a lack of pediatric population-based interventions. The short duration of most programs limits the possibility of evaluating the effectiveness of interventions [27]. Schools may be a key setting for programming where health status indicators, such as body composition, chronic disease risk factors and fitness, can all be positively impacted. Engagement in physical activity is a critical intervention in obesity prevention. Programs require sustained long-term resources to facilitate comprehensive evaluation that will ascertain if long-term impact such as sustained normal weight is maintained. Furthermore, involving stakeholders in program design, implementation and evaluation is crucial to the success of interventions, helping to ensure that needs are met [27]. Further research is required to understand the merits of the forms in which interventions are delivered and in which circumstances they are effective. There is a critical need for the development of consistent indicators to ensure that comparisons of program outcomes can be made to better inform best practice.

Age is a major factor that influences the specific strategies chosen helping the overweight and obese patient to control his/her weight. It is clear that at preschool age, prevention is more important than treatment, but later on treatment becomes necessary. In pre-school children it is easier to set up a treatment involving the family, in adolescents this approach will be less successful. We also have the impression that learning to adopt a healthy lifestyle, comprising of both diet and exercise, during childhood is easier than attempting to change these factors later in life [2,9,10,13]. Early treatment prevents the progression of overweight to severe obesity and related cardio-vascular risk factors. It is fundamental to continue to a full program of prevention and treatment rather than solely diet or only physical activity. It will be only with lifestyle changes that we could have the “unique” opportunity of controlling the “obesity epidemic”. 

## Future Perspectives

- Given the magnitude of the childhood obesity problem, pediatricians and other health care providers need to take a major role in the care of health of the obese child.
- Successfully treating obesity will require a major shift in pediatric care.
- The most effective way to prevent complications of obesity in teenagers is to introduce and reinforce healthy behavior and lifestyle early in childhood.
- More clinical studies on the efficacy of specific prevention and treatment programs; and the effort to move from efficacy to broad effectiveness are needed.

## Executive Summary

- Evaluating an obese child should begin with:
  - Detailed medical examination
  - Assessment of diet
  - Assessment of physical activity and behaviors that are linked to obesity
  - Appraisal of the degree of obesity and its associated metabolic complications
- Assessment of obesity/adiposity:
  - Body composition assessment/measurements: from height and weight, BMI (kg/m^2^), skinfold thickness and waist circumferences.
  - Assessment of fat, fat free mass, and bone mineral content using laboratory techniques (*i.e.*, BIA, Dual energy X-ray Absorptiometry (DXA), computed tomography (CT), Magnetic Resonance Imaging (MRI).
- Assessment of Obesity – related diseases:
  - Laboratory tests, (glucose, insulin, lipid profile, thyroid function, appropriate hormones, hepatic function), blood pressure assessment
- Specialized treatments – programs and interventions
  - Dietary components
  - Physical exercise
  - Pharmacologic approaches
  - Surgical approaches
  - Primary and specialized care

## Comments

It is important to establish objective evidence of the beneficial impact of any preventive and/or treatment program. To stop the epidemic of childhood obesity, it is mandatory to act at different levels including medical, social, educational and political [9]. A broad range of action would include establishing good nutrition education campaigns, regulating marketing of junk food, eliminating energy-dense food and sodas from being sold in schools and strongly promoting physical activity. 

Ultimately, we reemphasize the importance of early intervention in preventing the medical and psychosocial *sequelae* of obesity, as well as the persistence of obesity into adulthood. This underlines the need for increased awareness and identification of obesity in the primary care setting, especially among younger children.
